# Effects of Different Packaging Types and Storage Periods on Physicochemical and Antioxidant Properties of Honeys

**DOI:** 10.3390/foods13223594

**Published:** 2024-11-10

**Authors:** Yusuf Yiğit, Suzan Yalçın, Esin Ebru Onbaşılar

**Affiliations:** 1 Department of Hotel, Restaurant and Catering Services, Vocational School of Datça Kazım Yılmaz, Muğla Sıtkı Koçman University, Muğla 48900, Turkey; yusufyigit@mu.edu.tr; 2 Department of Food Hygiene and Technology, Faculty of Veterinary Medicine, Selçuk University, Konya 42003, Turkey; 3 Department of Animal Husbandry, Faculty of Veterinary Medicine, Ankara University, Ankara 06110, Turkey; onbasilar@ankara.edu.tr

**Keywords:** honey, physicochemical properties, antioxidant properties, packaging, storage period

## Abstract

Preserving the nutritional value of honey without compromising its properties until consumption is crucial. However, different types of honey may respond differently to packaging and storage conditions. This study aimed to assess the effects of different packaging materials (tin cans, light-colored glass jars, and dark-colored glass jars) and storage durations (initial, 6 months, and 12 months) on the physicochemical and antioxidant properties of pine, flower, and thyme honey. Nine samples were collected to conduct study on the three different types of honey. Honey samples were packaged in these materials and analyzed at the start, after 6 months, and after 12 months. The results showed that the moisture, proline content, sugar, total oxidant status (TOS), and oxidative stress index (OSI) levels were unaffected by honey type. Over time, there was a decrease in moisture, pH, proline content, diastase number, sugar, total phenolic content (TPC), total antioxidant status (TAS), and catalase activity, alongside an increase in the electrical conductivity, hydroxymethylfurfural (HMF), free acidity, TOS, and OSI levels. The packaging type did not influence the moisture, pH, electrical conductivity, proline content, diastase number, sugar, HMF, TPC, TAS, TOS, OSI, or catalase activity levels. The findings suggest that storing pine, flower, and thyme honey in light- or dark-colored glass jars or tin cans for 12 months does not significantly impact these properties. However, given the reduction in TPC and TAS across all honey types and packaging methods after 12 months, further studies are needed to explore ways to maintain honey quality in this regard.

## 1. Introduction

Honey, a unique natural product produced by bees (animal origin) using nectar from plants (plant origin), has been one of the most important nutrients for humans for centuries, highlighting its dual source and significance. It is recognized as a significant animal product that can be consumed directly by humans without any processing [1]. Honeys produced in greater abundance are from *Apis mellifera* species and are classified according to their origin: blossom or honeydew [2,3]. While blossom honeys are produced from the nectar of blossom, honeydew honeys are characterized as a product obtained from the exudates of plants or plant-sucking insects [2]. In Turkey, pine honeydew honey is the most well-known honeydew type, though other varieties like oak, fir, and cedar also exist [4]. 

The major constituents of honey are water and sugars, with a range of 80–85% and 15–17%, respectively [5]. The sugars commonly found in honey can be classified into two types: reducing sugars, which have a free aldehyde or ketone group that acts as a reducing agent, and non-reducing sugars, which do not. The most prevalent sugars in honey are reducing sugars, primarily fructose and glucose [6,7]. The minor constituents include enzymes such as α-glucosidase, β-glucosidase, α-amylase, glucose oxidase, invertase, diastase, catalase, proteases, and glucosylceramidase, along with proteins, amino acids, minerals, organic acids, water-soluble vitamins, lipids, pollen grains, phytochemicals, waxes, volatile organic compounds, and final products from the Maillard reaction, including various furfural derivatives and melanoidins [8,9].

The quality of honey is determined by its chemical, physical, microscopic, and organoleptic properties, which are affected by its plant origin, sugar composition, pH, and mineral content [10]. Furthermore, external factors such as climate, processing, packaging, and storage conditions play a vital role in maintaining honey’s quality [11].

As with all other foods, the correct selection of storage conditions for honey affects its quality [12]. Since glass jars do not engage in chemical reactions, storing honey in glass jars is known to be a successful method [13]. According to the Codex Alimentarius international food standards, honey should be stored in a cool, dry environment, ideally at temperatures below 20 °C, while also protecting it from moisture and direct sunlight. Similarly, the Turkish Standards Institution advises storing honey at temperatures not exceeding 25 °C [14]. When honey is exposed to the direct sunlight, the activity of the glucose oxidase enzyme is inhibited, causing the honey to lose its antibacterial properties. It has been emphasized that the ratio of water to glucose in honey affects the tendency to crystallize and that this tendency starts to accelerate at temperatures below 15 °C. If the glucose-to-water ratio is less than 1.7, the honey does not crystallize, whereas if the ratio is greater than 2.1, crystallization occurs more quickly. The processes applied to crystallized honey also affect its quality [15,16]. 

In honey, the levels of hydroxymethylfurfural (HMF) serve as an important quality and safety parameter, as high levels suggest a decrease in honey’s nutritional value and an increase in its potential toxicity [17,18]. Most previous studies have reported HMF’s negative effects on human health, including cytotoxicity toward mucous membranes, skin, and the upper respiratory tract, as well as mutagenicity, chromosomal aberrations, and carcinogenicity in humans and animals. It is not a direct mutagen but is converted into its active metabolite, 5-sulfo-oxymethylfurfural, which causes DNA damage in organs like the kidney and colon. However, recently, HMF was also shown to exhibit positive effects, such as antioxidative, anti-allergic, anti-inflammatory, anti-hypoxic, anti-sickling, and anti-hyperuricemic properties [19]. The amount of HMF in honey, in particular, results from the dehydration of hexoses in an acidic environment or Maillard (non-enzymatic browning) reactions and affects the chemical properties of honey, such as sugar content, total acidity, pH, and mineral content [9,20]. 

Diastase activity in honey is an important indicator of its freshness and quality, as diastase enzymes, particularly amylase, break down starch into simple sugars. Most studies have consistently shown a decreasing trend in diastase activity during storage, suggesting that the enzyme is sensitive to aging and environmental conditions. The reduction in diastase activity is primarily caused by the denaturation of the enzyme, which occurs due to prolonged storage and exposure to varying temperatures. Overall, the decline in diastase and other enzymatic activities highlights the importance of proper honey storage to maintain its enzymatic and nutritional integrity [9]. The diastase number is used to assess the freshness and authenticity of honey. A low diastase number suggests that the honey has been overheated, stored for too long, or adulterated [21].

In addition to the importance of legal compliance, the honey packaging industry is to ensure that the product obtained with great efforts reaches the consumer in a way that does not cause a decrease in its quality. Packaging materials protect food from degradation by providing various mechanisms, such as avoiding entry to the product, odor transmission prevention, and the conservation of an internal packaging environment [22]. Different containers, including tin cans, clay jars, and glass jars, are used for honey storage depending on the region, cost-effectiveness, and availability. The key factors to a quality end product are the proper packaging and storage of raw materials. 

Given the importance of maintaining honey’s physicochemical and antioxidant properties during storage [9], there is a need for further investigation into how different packaging materials and storage durations affect honey quality. Although research exists on the effects of temperature and storage conditions on various honey types [3,11,17,23,24,25], limited studies have compared the impact of packaging and storage on specific honey varieties like pine, flower, and thyme honeys. This study seeks to address this gap by analyzing the effects of different packaging materials (tin cans, light-colored glass jars, and dark-colored glass jars) and storage periods (initial, 6 months, and 12 months) on the physicochemical and antioxidant properties of these honey types obtained from the Aegean region. The findings of this research will provide valuable insights into the optimal packaging and storage conditions for preserving honey’s quality, particularly in relation to HMF levels, diastase activity, and antioxidant capacity. 

## 2. Materials and Methods

### 2.1. Honey Collection and Experimental Groups

The pine, flower, and thyme honey samples used in this study were obtained from the Muğla and Datça of the Aegean region directly from beekeepers. In total, nine samples were collected to conduct research on the three different types of honey. To determine the effects of different packaging materials—such as tin cans, light-colored glass jars, and dark-colored glass jars—on various types of honey, three samples of 400 g each were taken from each honey variety. This resulted in a total of 27 samples for analysis. Honeys were tightly closed to prevent contact with air and stored in a dark environment at room temperature (20–25 °C) until analysis. To determine the effect of storage time on 3 different types of honey, analyses were carried out on the first day after packaging, at the end of the 6th month, and at the end of the 12th month.

### 2.2. Determination Physicochemical Properties of Honey

#### 2.2.1. Moisture and pH Analysis

The moisture content of the honey samples was measured using a refractometric method [26]. A 1 g honey sample was placed in the glass chamber of a refractometer (Greinorm, Greiner Glasinstrumente, Lemgo, Germany). The moisture content was determined at 20 °C using a calculation chart. For pH analyses, 10 g honey sample was dissolved in 75 mL of distilled water, and the pH was measured using a pH meter (Crison Basic 20+ model, Crison Instruments SA, Barcelona, Spain) [26].

#### 2.2.2. Electrical Conductivity, Free Acidity, Proline Content, and Total Sugar Analysis

A 20 g honey sample was dissolved in 80 mL of water. The electrical conductivity was measured using a conductometer (Wtw cond7110, Weilheim, Germany) and expressed in μS/cm [26]. For free acidity analyses, 10 g honey sample was dissolved in 75 mL of water. A few drops of phenolphthalein indicator were added, and the solution was titrated with 0.05 N NaOH. The result was calculated in meq/kg [26].

Proline content analysis was conducted according to the method established by the International Honey Commission [27]. A 5 g honey sample was dissolved in 100 mL of distilled water. From this solution, 0.5 mL was taken and placed into test tubes. Then, 1 mL of formic acid and 1 mL of ninhydrin solution were added and mixed for 15 min. The tubes were kept in a boiling water bath for 15 min and then in a 70 °C water bath for 10 min. After the time had elapsed, 5 mL of a 1:1 (*v/v*) propanol–water mixture was added to each tube. The tubes were left for 45 min, and the absorbance was measured at 510 nm using a double-beam spectrophotometer (PG Instruments Ltd. T80 UV/VIS, Lutterworth, UK) against a blank. The proline content was expressed as mg/kg.

The total sugar content (fructose + glucose) of the honey samples was determined using a High Performance Liquid Chromatography (HPLC)-refractive index detector according to the method recommended by the International Honey Commission [27].

#### 2.2.3. Diastase Analysis

A 10 g honey sample was placed into a 100 mL volumetric flask and diluted with distilled water to the mark. The solutions were added to test tubes in different volumes, followed by the addition of starch solution. The tubes were then kept in a 40 °C water bath for 1 h. After the incubation period, the tubes were examined, and the first tube showing a blue color was taken as the endpoint [28]. The results were expressed as diastase number.

#### 2.2.4. Hydroxymethylfurfural (HMF) Analysis

The HMF content of the honey samples was determined according to the method recommended by the International Honey Commission [27]. The HPLC-UV detector (Agilent 1260 Infinity, Agilent Technologies, Santa Clara, CA, USA) with a column of C_18_-reversed phase material was used. The flow rate was 1.0 mL/min, and the injection volume was 20 µL. The mobile phase consists of a water–methanol mixture (90:10, *v/v*), both of HPLC grade. The HMF content in the honey was expressed in mg/kg [27].

### 2.3. Determination of Antioxidant–Oxidant Status

A 0.1 mL honey sample was taken and placed into 2 mL Eppendorf tubes, to which 1.4 mL of ethanol was added. The tubes were vortexed for 5 min. The samples were then centrifuged at a gravitational force of 25,152× *g* for 10 min, and the supernatants were transferred to clean Eppendorf tubes. The total antioxidant status (TAS) and total oxidant status (TOS) of these ethanol extracts were measured using commercial kits (Rel Assay Diagnostics, Gaziantep, Turkey) on a chemistry analyzer (Mindray, BS-400, Shenzhen, China). The TAS was measured at a wavelength of 660 nm and expressed as mmol Trolox Equivalent/kg, while the TOS was measured at 530 nm and expressed as μmol H_2_O_2_ Equivalent/kg. The values obtained from the honey samples were multiplied by the dilution factor to calculate the TAS, TOS, and oxidative stress index (OSI) values in the honey samples, as reported by Ramay and Yalçın [29].

### 2.4. Determination of Catalase and Total Phenolic Content (TPC) Activity

The catalase activity in the honey samples was measured at a wavelength of 405 nm using a commercial kit (Rel Assay Diagnostics, Gaziantep, Turkey) on a chemistry analyzer (Mindray, BS-400, Shenzhen, China) and expressed as U/kg [30].

The total phenolic content in the honey samples was determined using the Folin–Ciocalteu method [31,32], with a spectrophotometer (Rel Biochem, Rel Assay Diagnostics, Gaziantep, Turkey). Gallic acid was used as the reference standard for the TPC analysis. Calibration curves were generated using gallic acid solutions at concentrations ranging from 0 to 300 mg/L. The resulting calibration curve (y = 0.0027x + 0.0293, where y represents absorbance and x represents the concentration of gallic acid) had a coefficient of determination (r^2^) of 0.9973, indicating excellent linearity within the tested concentration range. The TPC of the samples was expressed as gallic acid equivalents (mg GAE/100 g).

### 2.5. Statistical Analysis

The statistical analysis of the samples was performed using IBM-SPSS 22 (SPSS Inc., Chicago, IL, USA).

The study examined the effects of different honey types and packaging methods on the physicochemical properties and antioxidant parameters over time using “repeated measures ANOVA (general linear models)”. Specifically, the interaction between the honey type and packaging method was analyzed to assess whether the combination of these factors influenced the outcomes. Additionally, changes within each group (honey type or packaging method) over time were tested to determine how these factors evolved throughout the study period. The interaction between the honey type and packaging type groups was analyzed, and any within-group differences over time were tested. The estimated marginal mean values and standard error of mean for the variables were calculated. After performing repeated measures ANOVA, a post hoc test, Least Significant Difference was used to determine which specific means are significantly different from each other.

The correlation between physicochemical properties and antioxidant parameters during the study periods was assessed using Spearman’s rho correlation analysis. 

A *p*-value of less than 0.05 was considered statistically significant for all analyses.

## 3. Results

**Figure 1:**
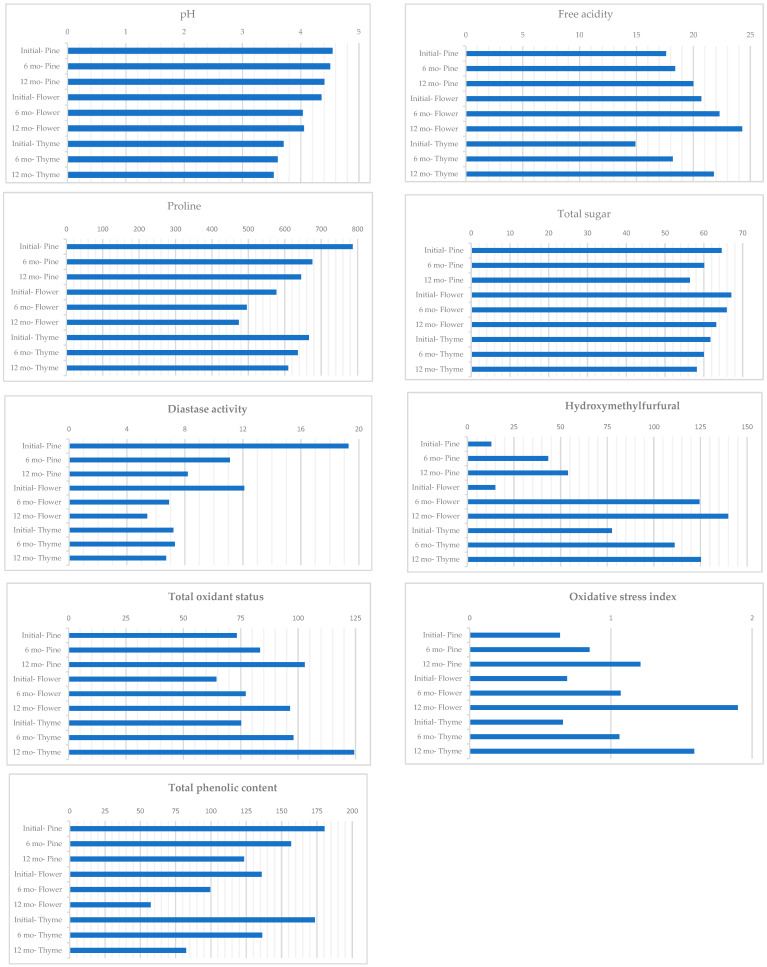
Differences in pH, free acidity, proline, total sugar, diastase activity, hydroxymethylfurfural, total oxidant status, oxidative stress index, and total phenolic content were observed based on conditions, including honey type and storage period.

The pH, electrical conductivity, free acidity levels, diastase activity, HMF, TAS, catalase activity, and TPC varied significantly depending on the type of the honey (*p* < 0.05, Table 1 and Table 2). The type of packaging only influenced the free acidity levels of the honey (*p* < 0.05, Table 1). The storage duration significantly affected all the examined properties (*p* < 0.05, Table 1 and Table 2). As the storage time increased, the levels of moisture, pH, proline content, diastase activity, sugar, TAS, catalase, and TPC decreased, while the electrical conductivity, HMF, free acidity, TOS, and OSI levels increased. The interaction between the honey type and storage time was significant for most properties examined (*p* < 0.05, Figure 1). An interaction between the packaging type and storage period was observed for free acidity levels (*p* < 0.05, Table 1).

Moisture content did not vary according to the honey type or packaging, but it was observed to decrease during the storage period (Table 1). 

Pine honey had the highest pH value, while thyme honey had the lowest. The pH was highest in fresh samples, but decreased by the 6th month and remained stable until the 12th month. When examining the interaction between time and honey type, a significant decrease over time was observed only in flower honey (Table 1, Figure 1). 

Pine honey had nearly three times higher electrical conductivity compared to the other honey types. When examining all honey types, it was observed that electrical conductivity increased with storage time (Table 1).

Free acidity varied across all three study groups. Among the honey types, flower honey had higher free acidity than others. Among the packaging types, light-colored glass had the lowest free acidity. Free acidity increased over time. When the interaction between time and honey type was analyzed, flower honey exhibited the highest free acidity at the 12th month. In the interaction between time and storage container, tin containers showed the highest acidity at the 12th month (Table 1, Figure 1).

The proline content declines significantly over time. No significant difference was detected in the proline content according to honey type. Additionally, there was no significant interaction between the packaging type and storage time by repeated measures variance analysis. However, when analyzing the interaction between the honey type and storage duration, pine honey had the highest level in the initial period and flower honey had the lowest level at the 12th month (Table 1, Figure 1).

Diastase activity varies depending on the type of honey, with pine honey showing the highest activity and thyme honey the lowest. Over time, diastase activity decreased significantly, particularly in pine and flower honeys. Interestingly, thyme honey does not exhibit a significant change in diastase activity with prolonged storage (Table 2). Diastase activity and TPC were highest in the initial pine honeydew honey, while the lowest levels were observed in flower honey at the 12th month (Table 2, Figure 1). 

Among the three types of honey studied, pine honey had the lowest HMF value. The HMF value showed a significant increase with storage time. When analyzing the interaction between time and honey type, it was observed that this increase was statistically significant in flower honey during the first six months. HMF values were highest in the 12-month flower, 12-month thyme, and 6-month flower honeys, but lowest in the initial pine and initial flower honeys (Table 2, Figure 1). 

Among the three types of honey, flower honey had the lowest TAS value. When these honey types were examined together, a decrease in the TAS levels was observed over time. However, this decrease did not show any statistically significant differences concerning time, honey type, or storage conditions (Table 2).

While the TOS and OSI levels did not vary according to honey type or packaging type, an increase was observed over time. The TOS was highest in the 12-month thyme honey, while it was lowest at the start for all three types of honey (Table 2, Figure 1).

Catalase levels were higher in pine honey compared to flower honey, and it was observed that catalase levels decreased over time (Table 2). 

The TPC was highest in pine honey and lowest in flower honey, with a noted decrease in TPC levels over time. When examining the interaction between time and honey type, it was found that all honey groups showed a decrease within themselves, with the highest level recorded in pine honey at the baseline sample and the lowest level observed in flower honey at the 12th month (Table 2, Figure 1).

Correlations among parameters showed notable changes depending on the storage period (Supplementary File). The correlation between diastase activity and both the pH and electrical conductivity in the honey was highly significant at the beginning and 6th months after storage (*p* < 0.001). However, 12 months after storage, the correlation with pH was no longer significant, and the correlation with electrical conductivity was moderately significant (*p* = 0.003). The correlation between the TPC and TAS remained significant across all periods (*p* < 0.001). While no relationship was found between the initial total sugar and proline content or diastase, a negative relationship was observed between total sugar and diastase and a positive relationship between total sugar and HMF at both the 6th and 12th months (Supplementary File).

## 4. Discussion

The findings of this study underscore the influence of honey type, packaging, and storage time on various physicochemical properties and bioactive components of honey. Notably, pine honeydew honey exhibited the highest levels of pH, electrical conductivity, and diastase activity, while thyme honey showed lower concentrations of these attributes. Additionally, the levels of moisture, proline, and sugars remained relatively constant across the different honey types examined. However, storage conditions significantly impacted these properties, with increases in electrical conductivity, HMF, and free acidity, alongside decreases in the TPC, TAS, and catalase activity over time. This suggests that prolonged storage, regardless of packaging type, can lead to the degradation of beneficial compounds in honey, necessitating further investigation into optimal storage conditions to maintain honey quality.

Moisture is a crucial parameter in determining the quality of honey. The amount of water present in honey affects its stability against fermentation and granulation. High moisture levels can lead to undesirable fermentation and the formation of acetic acid during storage. The moisture content in honey should not exceed 20% [21]. Juan-Borrás et al. [33] reported that the moisture content in thyme honey was 19.5%. In the present study, the moisture level was found to be in the same range among honey types, at 16.0–16.2%. In this study, it was observed that the moisture content decreased as the storage period extended when different types of honey were stored at room temperature for 12 months in various packaging types. Similarly, Minhas [34] reported that the storage had a significantly decreasing effect on the moisture content of honey packaged in plastic jars and poly pack pouches. The moisture content of honey decreased to 16.41 and 16.63% in plastic jars and polypack pouches, respectively, from an initial 18.1% after 12 months of storage [34]. Seraglio et al. [24], however, did not find significant differences between the moisture content before and after honey storage. Contrary to that, da Silva et al. [35] established an increase in the moisture content before and after honey storage, and the differences were statistically significant. Different results may be the consequence of the influence of numerous factors, like the environment, the time of harvest, and the level of honey maturation attained in the hive [12]. It has been noted that exceeding the permissible moisture levels could increase the risk of spoilage due to fermentation, thereby reducing the quality of honey [36,37]. Additionally, it was observed that honey and packaging type were not effective in decreasing the moisture level over time. In this case, the increase over time changes only as the package comes into contact with air.

The pH of honey is an important parameter as it affects the texture, stability, and shelf life of honey [38]. Honey is acidic in nature, with a pH ranging from 3.4 to 6.1 [39]. The degree of acidity in honey can depend on the amount and type of free acids present [40]. The pH of the nectar, the soil characteristics of the region where the honey is collected, and the flowering period are also factors affecting the pH of honey. In this study, the pH values of honey were found to range from 3.61 to 4.48, with thyme honey being more acidic (average pH of 3.61) and pine honeydew honey being less acidic (pH = 4.48). The findings of this study indicate that the amount of free acidity in honey samples is proportional to their degree of acidity. The study also found that in various honey types stored for 12 months in different packaging types, the pH level decreased, and the free acidity increased as the storage period progressed. These findings are consistent with the results of some studies [25,34,37,41]. This change may result from reactions between various compounds in honey, the amount of glucose, and the concentration of ions present in honey [25,37]. Glucose in honey is converted into gluconic acid by the action of D-glucose oxidase. This reaction leads to the formation of hydrogen peroxide, an important antimicrobial agent in honey [25,42]. The production of acid in honey can also occur through fermentative processes facilitated by microorganisms [25,43,44]. The decrease in pH during storage may be due to the reduction in some solid substances and the formation of salts and bases, resulting in the observed changes in pH. Since the change in thyme honey according to storage time was not statistically significant, an interaction was observed between the storage time and packaging type in terms of the pH values. 

The free acidity of honey is primarily due to the presence of organic acids, particularly gluconic acid, which exists in equilibrium with lactones or internal esters and some inorganic ions like phosphates [45]. Free acidity is an important quality parameter that influences both the flavor and stability of honey. The botanical origin of honey significantly affects its acidity, as aromatic acids play a key role in contributing significantly to its flavor [40]. Elevated acidity can be a sign of sugar fermentation (fructose, glucose) or spoilage, both of which negatively impact the taste and quality of honey. In this study, the free acidity of honey samples collected from the Aegean region ranged from 18.3 to 22.5 meq/kg, which is below the maximum allowable value set by Turkish standards, as well as the Codex Alimentarius and European Union standards. These standards do not specify a minimum value, as the natural acidity of honey can vary based on its floral source [2,14,21]. The highest free acidity was observed in flower honey, with a value of 22.5 meq/kg. Faleye et al. [40] reported free acidity values between 27 and 40 meq/kg in Nigerian polyfloral honeys, which are higher than the findings of this study. Pirdawd [46] reported free acidity values ranging from 18.67 to 37.09 meq/kg, with an average of 24.62 meq/kg, in honey collected from the Erbil region. Zivkov Balos et al. [12] reported free acidity values ranging from 1.5 to 29.0 meq/kg in honey collected from Northern Serbia, with an average of 13.61 meq/kg in flower honey and 19.33 meq/kg in forest honey. The reason for the low free acidity of honey in light-colored jars should be investigated in more detail. Seraglio et al. [24] and Da Silva et al. [35], found that free acidity increased during storage time. The increase in free acidity as the storage period increases was highest in flower honey than in other honeys, which caused an interaction between the honey type and storage time.

There are significant differences in the electrical conductivity between floral and honeydew honeys. In the study, the electrical conductivity of flower and thyme honeys was found to be 397.1 and 365.6 μS/cm on average, respectively, while pine honeydew honey exhibited a higher conductivity of 937.2 μS/cm. These results are within the range of national and international standards. The maximum allowed value for electrical conductivity in the national and international standards [2,14,21] is reported to be ≤0.8 mS/cm for floral and honeydew honeys with certain exceptions, particularly for honeydew honey, which can exceed 0.8 mS/cm due to its natural higher mineral content. Faleye et al. [40] reported electrical conductivity in floral honeys ranging from 50 to 470 μS/cm. Similarly, Zivkov Balos et al. [12] found that the electrical conductivity of honeys collected from Northern Serbia ranged from 180 to 1120 μS/cm, with an average of 460 μS/cm in floral honeys and 1120 μS/cm in honeydew honeys. Electrical conductivity increased with storage time. Similarly to the research findings, Bhalchandra et al. [41] determined that the electrical conductivity of fresh honey, which was 0.29 mS/cm, was 0.98 mS/cm after 24 months. Electrical conductivity is one of the most important characteristics in determining the physical properties of honey, providing information about the source of the honey (such as flower or forest origin or the nectar source) [40]. The electrical conductivity of honey is dependent on the concentration of the organic acids, proteins, certain complex sugars, polyols, and mineral salts present in the honey [41,47], and it is also an indicator of honey freshness. 

Proline, one of the highest amino acid found in high levels in honey, indicates the freshness of honey [47,48]. Proline is also an indicator of honey maturity and whether the honey has been adulterated with sugar [49,50,51]. If the proline level falls below 180 mg/kg, it suggests sugar adulteration [41]. In this study, while the proline content did not vary significantly according to the honey type or packaging type, it decreased as storage time progressed. Consistent with the findings of this study, Bhalchandra et al. [41] reported that the proline content in fresh honey decreased from 1026.23 mg/kg to 840.12 mg/kg after 24 months of storage. In the first six months, the proline content decreased by 27.66 mg/kg, and by 12 months, it had decreased by 121.85 mg/kg. The decrease in the proline level as the storage period increases was less in thyme honey than in other honeys, which caused an interaction between the honey type and storage time. 

Carbohydrates are the main components of honey, making up approximately 95% of its dry weight. The composition of sugars in honey generally depends on the type of flora in the region, the source, and the environmental conditions. Fructose and glucose are among the most important carbohydrates found in honey. In the study, the level of sugars (fructose + glucose) in fresh honeys was found to be above 60%. However, with storage, this level decreased to an average of 59.3% after 12 months. This decline in the total sugar content during storage indicates the conversion of sucrose into glucose and fructose. The decrease in the total sugar as the storage period increases was less in thyme honey than in other honeys, which caused an interaction between the honey type and storage time. 

Diastase activity, measured by the diastase number, reflects the role of natural enzymes α-amylase and β-amylase in breaking down starch and reducing honey’s viscosity. This activity is influenced by factors such as the nectar source, regional geography, and local flora [46]. While no significant interaction was observed between the honey type and packaging in this study, diastase activity decreased over time during storage, consistent with previous research [34,41]. However, thyme honey showed a slower decline in diastase activity compared to other types, leading to a significant interaction between the honey type and storage time. This might be due to its higher concentrations of phenolic compounds, antioxidants, and bioactive substances. These compounds may help stabilize the diastase enzyme and preserve proline, contributing to thyme honey’s greater resistance to enzyme degradation during storage [52,53,54,55]. 

In this study, the HMF levels in fresh pine and flower honeys were below 40 mg/kg in the initial samples, in accordance with Turkish, European Union, and Codex Alimentarius standards [2,14,21]. However, thyme honey had a higher HMF level of 77.6 mg/kg, still within the acceptable limit of 80 mg/kg for honeys from tropical regions, which may be attributed to the warm climate of the Muğla region. 

As the storage time increased, the HMF levels rose, especially in flower honey, leading to a significant interaction between the honey type and storage duration. In this study, while the packaging type did not significantly affect the HMF levels, a clear increase was observed over storage time, consistent with findings by Wang et al. [56], who reported HMF increases of 102–181% in alfalfa honey stored at room temperature. Honey naturally contains sugars like fructose, glucose, and sucrose, with HMF produced from the degradation of these sugars. HMF is a key indicator of honey freshness, influenced by the storage conditions, packaging, and type of flora [10,17,46]. Key factors contributing to HMF formation include heating during honey processing, which reduces viscosity and prevents crystallization, as well as the physicochemical properties of honey, such as pH, acidity, water activity, and mineral content. HMF formation is accelerated at a low pH, higher temperatures, longer storage durations, and with higher moisture content. Additionally, the fructose-to-glucose ratio significantly affects HMF production, as fructose is more reactive than glucose. 

HMF levels increase as honey ages [20], and its formation is a general indicator of Maillard reactions, which occur during long-term storage, leading to changes in the honey’s composition. These reactions, along with the caramelization of carbohydrates and the breakdown of fructose in honey’s acidic environment, result in the production of HMF and other compounds [57]. While Maillard reaction products may act as antioxidants [58], no significant correlation was found between the HMF and antioxidant parameters, such as the TAS, TOS, OSI, or TPC, at the start or during storage in our study. Similar studies also did not find a link between HMF and antioxidant activity [52,56]. 

Interestingly, Monggudal et al. [37] reported an increase in the TPC when honey was stored in glass jars covered with aluminum foil at 4 °C, suggesting the potential formation of new phenolic compounds or by-products due to enzyme reactions during storage.

The determination of phenolic compounds in honey samples is of great importance [59]. In this study, the TPC in the honeys was found to range from 97.7 to 153.6 mg GAE/100 g. Faleye et al. [40] reported TPCs in floral honeys ranging from 108 to 197 mg GAE/100 g. Koç et al. [60] found the TPC in Denizli thyme honeys to range between 69.8 and 81.3 mg GAE/100 g, with an average of 75.9 mg GAE/100 g. Kıvrak et al. [61] reported an average TPC of 106.46 mg GAE/100 g in thyme honey. Pirdawd [46] found TPC contents in honeys collected from the Erbil region to range between 57.30 and 155.47 mg GAE/100 g. Meda et al. [62] reported TPCs in honeys ranging from 32.59 to 114.75 mg GAE/100 g and found higher TPCs in honeydew honeys compared to floral honeys. In this study, no differences were observed in TPCs according to packaging type, but the TPC decreased as storage time progressed. Similarly, Wang et al. [56] reported a significant decrease of 25% in the TPC in alfalfa and buckwheat honeys stored in dark glass jars for six months, with a 17% decrease observed in buckwheat honey. They also noted that storage in light-colored glass jars did not affect the TPC.

The antioxidant activity of honey is influenced by the presence of Maillard reaction products, enzymes, phenolic compounds, flavonoids, organic acids, amino acids, peptides, ascorbic acid, and carotenoid-like compounds [63]. In Iraq’s Erbil region, honeys with high levels of total phenolic compounds also exhibited high antioxidant activity [46]. Similarly, in this study, a strong and significant correlation was found between the TPC and TAS (r = 0.64, *p* < 0.001). The study revealed that pine honeydew honey and thyme honey had the highest levels of TAS and catalase activity, while flower honey had the lowest levels. No significant differences in the TAS and catalase levels were observed between honey stored in glass jars and those stored in tin containers. However, as the storage period progressed, there was a significant decrease in the TAS and catalase levels and a significant increase in the TOS and OSI levels (*p* < 0.001). The TOS and OSI levels did not vary with the type of honey or packaging. Similar findings were reported by Wang et al. [56], who noted a decrease in the antioxidant capacity by 32% and 49% in alfalfa and buckwheat honeys, respectively, during storage, regardless of the packaging type (light and dark plastic containers and light and dark glass jars). Consistent with this study, Wang et al. [56] also found a strong and significant correlation between the TPC and antioxidant capacity in honey.

When all three types of honey were analyzed together, it was found that the relationship between the HMF and total sugar levels becomes significant from the 6th month of storage, while the relationship between the total sugar and moisture was only significant in the 6th month of storage. Additionally, the relationships between free acidity and electrical conductivity, as well as between TAS and free acidity, were found to be insignificant after the 6th month of storage. The relationship between the catalase and TPC was significant only in the 12th month of storage. These findings suggest that the relationships among honey characteristics vary depending on the duration of storage, likely due to the differing properties of each honey type.

As a limitation, the number of honey samples analyzed may not fully represent the diversity of honey types available in different regions. Future studies could benefit from a larger and more varied sample size to strengthen the generalizability of the findings. In addition, although the study assessed the impact of different packaging types and storage durations, it did not explore a comprehensive range of environmental conditions such as temperature fluctuations, humidity levels, or exposure to light, all of which could significantly affect honey quality. The study’s strengths include its comprehensive approach, examining multiple honey types and packaging methods, which enhances the understanding of how these factors interact to influence honey quality. Additionally, the correlation observed between the TPC and TAS across various storage periods emphasizes the interconnectedness of honey’s antioxidant properties, providing a basis for future research on optimizing honey preservation techniques.

## 5. Conclusions

In conclusion, this study highlights the significant role of the honey type, packaging, and storage duration in determining the physicochemical characteristics and antioxidant properties of honey. The results indicate that pine honeydew honey consistently outperformed floral honey regarding pH, electrical conductivity, and diastase activity. In contrast, floral honey exhibited lower concentrations of these beneficial attributes, underscoring the importance of honey variety in determining its overall quality. Moreover, the investigation revealed that free acidity and HMF levels exhibited notable changes over time, indicating that prolonged storage can lead to degradation in these critical quality parameters. The observed decline in the TPC, TAS, and catalase activity further suggests that the health benefits associated with honey may diminish with extended storage periods. These findings highlight the need for the careful consideration of storage conditions and durations to preserve the bioactive components that contribute to honey’s therapeutic potential. Given these findings, further research should explore diverse storage methods and container types to maximize honey’s health benefits and maintain its quality over time. This investigation contributes to the broader understanding of honey as a functional food and supports the development of best practices for honey storage and consumption.

## Figures and Tables

**Table 1:** Moisture (%), pH, electrical conductivity (μS/cm), free acidity (meq/kg), proline content (mg/kg), and total sugar (%) values in the groups *.

Groups	Moisture	pH	Electrical Conductivity	Free Acidity	Proline	Total Sugar
**Honey type (HT)**						
Pine	16.0	4.48 ^a^	937.2 ^a^	18.6 ^b^	702.8	60.4
Flower	16.2	4.14 ^b^	397.1 ^b^	22.5 ^a^	515.6	65.4
Thyme	16.2	3.61 ^c^	365.6 ^b^	18.3 ^b^	637.4	60.0
**Packaging type (PT)**						
Tin cans	16.1	4.11	585.3	21.1 ^a^	614.4	61.9
Light-colored glass	16.1	4.06	550.5	17.7 ^b^	618.5	61.8
Dark-colored glass	16.1	4.06	564.2	20.6 ^a^	622.8	61.9
**Storage period (SP)**						
Initial	16.5 ^a^	4.20 ^a^	498.6 ^c^	17.7 ^c^	676.9 ^a^	64.5 ^a^
6 mo	16.2 ^b^	4.04 ^b^	561.7 ^b^	19.6 ^b^	602.6 ^b^	62.0 ^b^
12 mo	15.6 ^c^	3.99 ^b^	639.7 ^a^	22.0 ^a^	576.2 ^c^	59.3 ^c^
**SP × HT**						
Initial—Pine	16.4	4.54 ^a^	856.1	17.6 ^d^	787.0 ^a^	64.6 ^abc^
6 mo—Pine	16.1	4.50 ^ab^	932.2	18.4 ^cd^	676.1 ^ab^	60.1 ^cde^
12 mo—Pine	15.4	4.40 ^ab^	1023.3	20.0 ^bcd^	645.2 ^ab^	56.4 ^e^
Initial—Flower	16.4	4.35 ^b^	331.7	20.7 ^bc^	577.2 ^bcd^	67.1 ^a^
6 mo—Flower	16.3	4.03 ^c^	391.4	22.3 ^ab^	495.7 ^cd^	65.9 ^ab^
12 mo—Flower	15.8	4.05 ^c^	468.2	24.3 ^a^	473.8 ^d^	63.2 ^abc^
Initial—Thyme	16.7	3.70 ^d^	308.0	14.9 ^e^	666.6 ^ab^	61.7 ^bcd^
6 mo—Thyme	16.2	3.60 ^d^	361.3	18.2 ^cd^	636.1 ^bc^	60.0 ^cde^
12 mo—Thyme	15.7	3.53 ^d^	427.4	21.8 ^ab^	609.5 ^bcd^	58.2 ^de^
**SP × PT**						
Initial—Tin cans	16.5	4.25	497.4	18.2 ^cd^	676.7	64.5
6 mo—Tin cans	16.1	4.04	565.9	20.9 ^bc^	592.3	61.7
12 mo—Tin cans	15.5	4.05	692.4	24.3 ^a^	574.2	59.7
Initial—Light-colored glass	16.5	4.13	502.7	16.6 ^d^	677.2	64.5
6 mo—Light-colored glass	16.2	4.09	557.6	17.4 ^d^	605.5	61.8
12 mo—Light-colored glass	15.7	3.97	591.2	19.0 ^cd^	572.7	59.2
Initial—Dark-colored glass	16.5	4.21	495.7	18.3 ^cd^	676.9	64.4
6 mo—Dark-colored glass	16.2	4.00	561.6	20.6 ^bc^	610.0	62.5
12 mo—Dark-colored glass	15.7	3.95	635.3	22.8 ^ab^	581.5	58.9
**Overall Estimated mean ± SEM**	16.1 ± 0.1	4.08 ± 0.03	566.6 ± 10.3	19.8 ± 0.3	618.6 ± 30.9	61.9 ± 1.0
**Significance**	*p*	*p*	*p*	*p*	*p*	*p*
**Between subject effects**						
HT	0.290	<0.001	<0.001	<0.001	0.067	0.072
PT	0.876	0.661	0.400	0.001	0.994	0.999
HT × PT	0.323	0.821	0.285	0.140	1.000	0.999
**Within subject contrasts**						
SP	<0.001	<0.001	<0.001	<0.001	<0.001	<0.001
SP × HT	0.318	0.023	0.387	<0.001	<0.001	0.005
SP × PT	0.744	0.232	0.208	0.003	0.824	0.874
SP × HT × PT	0.319	0.733	0.038	0.876	0.852	0.997

* Repeated measures ANOVA (general linear model). estimated mean values were given. SEM: standard error of mean; ^a,b,c,d,e^ difference between values with different letters on the same column is statistically significant (pairwise comparison, LSD).

**Table 2:** Diastase activity, HMF (mg/kg), TAS (mmol Trolox Equivalent/kg), TOS (μmol H_2_O_2_ Equivalent/kg), OSI, catalase (U/kg), and TPC (mg GAE/100 g) values in the groups *.

Groups	Diastase Activity	Hydroxymethylfurfural	Total Antioxidant Status	Total Oxidant Status	Oxidative Stress Index	Catalase	Total Phenolic Content
**Honey type (HT)**							
Pine	12.9 ^a^	36.8 ^b^	10.0 ^a^	86.5	0.90	104.0 ^a^	153.6 ^a^
Flower	8.2 ^b^	93.1 ^a^	7.2 ^b^	79.3	1.22	90.8 ^b^	97.7 ^c^
Thyme	7.0 ^c^	104.7 ^a^	9.6 ^a^	99.2	1.10	97.2 ^ab^	130.8 ^b^
**Packaging type (PT)**							
Tin cans	9.3	85.9	8.9	87.2	1.05	94.9	124.6
Light-colored glass	9.4	75.8	9.3	88.5	1.02	94.0	129.5
Dark-colored glass	9.4	72.9	8.6	89.4	1.16	103.1	127.9
**Storage period (SP)**							
Initial	12.9 ^a^	35.2 ^c^	10.7 ^a^	71.0 ^c^	0.67 ^c^	108.9 ^a^	163.4 ^a^
6 mo	8.4 ^b^	93.0 ^b^	8.9 ^b^	86.2 ^b^	0.99 ^b^	97.6 ^b^	130.9 ^b^
12 mo	6.8 ^c^	106.4 ^a^	7.3 ^c^	107.9 ^a^	1.57 ^a^	85.5 ^b^	87.7 ^c^
**SP × HT**							
Initial—Pine	19.3 ^a^	12.9 ^d^	11.4	73.3 ^e^	0.64 ^e^	114.3	180.5 ^a^
6 mo—Pine	11.1 ^b^	43.4 ^cd^	10.1	83.4 ^bcde^	0.85 ^de^	104.0	156.8 ^b^
12 mo—Pine	8.2 ^c^	54.0 ^cd^	8.6	102.9 ^b^	1.21 ^c^	93.6	123.5 ^c^
Initial—Flower	12.1 ^b^	15.0 ^d^	9.3	64.4 ^e^	0.69 ^e^	101.8	136.0 ^c^
6 mo—Flower	6.9 ^cd^	124.5 ^ab^	7.2	77.2 ^cde^	1.07 ^cd^	93.1	99.6 ^d^
12 mo—Flower	5.4 ^e^	139.9 ^a^	5.2	96.5 ^bcd^	1.90 ^a^	77.6	57.3 ^e^
Initial—Thyme	7.2 ^cd^	77.6 ^bc^	11.4	75.2 ^de^	0.66 ^e^	110.6	173.7 ^ab^
6 mo—Thyme	7.3 ^cd^	111.2 ^ab^	9.4	98.0 ^bc^	1.06 ^cd^	95.6	136.3 ^c^
12 mo—Thyme	6.7 ^de^	125.3 ^ab^	8.1	124.4 ^a^	1.59 ^b^	85.3	82.4 ^d^
**SP × PT**							
Initial—Tin cans	12.8	35.1	10.5	71.1	0.68	109.2	160.6
6 mo—Tin cans	8.3	104.5	8.9	85.9	0.97	95.9	128.1
12 mo—Tin cans	6.6	118.1	7.4	104.5	1.49	79.6	85.0
Initial—Light-colored glass	12.8	35.2	11.0	70.7	0.64	103.9	169.1
6 mo—Light-colored glass	8.7	88.7	9.3	86.8	0.96	93.7	135.0
12 mo—Light-colored glass	6.7	103.5	7.5	107.9	1.46	84.3	84.5
Initial—Dark-colored glass	12.9	35.2	10.6	71.0	0.67	113.5	160.5
6 mo—Dark-colored glass	8.3	85.9	8.5	85.9	1.05	103.2	129.6
12 mo—Dark-colored glass	7.0	97.6	6.9	111.4	1.75	92.7	93.7
**Overall Estimated mean ± SEM**	9.4 ± 0.2	78.2 ± 10.7	9.0 ± 0.2	88.4 ± 4.3	1.07 ± 0.05	97.3 ± 1.6	127.3 ± 3.3
**Significance**	*p*	*p*	*p*	*p*	*p*	*p*	*p*
**Between subject effects**							
HT	<0.001	0.040	<0.001	0.187	0.053	0.013	<0.001
PT	0.901	0.873	0.407	0.977	0.521	0.061	0.822
HT × PT	0.964	0.979	0.218	0.843	0.792	0.563	0.962
**Within subject contrasts**							
SP	<0.001	<0.001	<0.001	<0.001	<0.001	<0.001	<0.001
SP × HT	<0.001	0.004	0.207	0.043	0.010	0.581	0.035
SP × PT	0.969	0.680	0.684	0.670	0.295	0.082	0.363
SP × HT × PT	0.995	0.881	0.381	0.284	0.355	0.422	0.626

* Repeated measures ANOVA (general linear model). estimated mean values were given. SEM: standard error of mean; ^a,b,c,d,e^ difference between values with different letters on the same column is statistically significant (pairwise comparison, LSD).

## Data Availability

The original contributions presented in this study are included in the article/Supplementary Materials, further inquiries can be directed to the corresponding author.

## Supplementary Materials

The following supporting information can be downloaded at https://www.mdpi.com/article/10.3390/foods13223594/s1. Supplementary File. Spearman rho correlation analysis of physicochemical and antioxidant parameters at the initial, 6th, and 12th month of the storage in the groups.

## References

[1] Crane E. (2004). A short history of knowledge about honey bees (Apis) up to 1800. Bee World.

[2] Codex Alimentarius Commission (2001). Revised Codex Standards for Honey CXS 12-1981. Rev 1(1987), Rev 2, FAO. https://www.fao.org/4/w0076e/w0076e30.htm.

[3] Bergamo G., Seraglio S.K.T., Gonzaga L.V., Fett R., Costa A.C.O. (2019). Physicochemical characteristics of bracatinga honeydew honey and blossom honey produced in the state of Santa Catarina: An approach to honey differentiation. Food Res. Int..

[4] Ünal S., Ayan S., Karadeniz M., Yer E. (2017). Some forest trees for honeydew honey production in Turkey. Sib. Lesn. Zurnal (Sib. J. For. Sci.).

[5] Khan S.U., Anjum S.I., Rahman K., Ansari M.J., Khan W.U., Kamal S., Khattak B., Muhammad A., Khan H.U. (2018). Honey: Single food stuff comprises many drugs. Saudi J. Biol. Sci..

[6] Kunz T., Lee E., Schiwek V., Seewald T., Methner F. (2011). Glucose—A reducing sugar? Reducing properties of sugars in beverages and food. Brew. Sci..

[7] Derebaşı E., Bulut G., Col M., Güney F., Yaşar N., Ertürk Ö. (2014). Physicochemical and residue analysis of honey from Black Sea region of Turkey. Fresenius Environ. Bull..

[8] Manickavasagam G., Saaid M., Lim V., Saad M., Azmi N.A.S., Osman R. (2023). Quality assessment and chemometrics application on physicochemical characteristics, antioxidant properties, and 5-HMF content of Malaysian stingless bee honey from different topographical origins. J. Food Sci..

[9] Manickavasagam G., Saaid M., Lim V. (2024). Impact of prolonged storage on quality assessment properties and constituents of honey: A systematic review. J. Food Sci..

[10] Fallico B., Arena E., Zappala M. (2009). Prediction of honey shelf life. J. Food Qual..

[11] Tornuk F., Karaman S., Ozturk I., Toker O.S., Tastemur B., Sagdic O., Dogan M., Kayacier A. (2013). Quality characterization of artisanal and retail Turkish blossom honeys: Determination of physicochemical, microbiological, bioactive properties and aroma profile. Ind. Crop. Prod..

[12] Zivkov Balos M., Popov N., Jaksic S., Mihaljev Z., Pelic M., Ratajac R., Ljubojevic Pelic D. (2023). Sunflower honey-evaluation of quality and stability during storage. Foods.

[13] Nascimento A.G.M., Toledo B.S., Guimarães J.T., Ramos G., da Cunha D.T., Pimentel T.C., Cruz A.G., Freitas M.Q., Esmerino E.A., Mársico E.T. (2022). The impact of packaging design on the perceived quality of honey by Brazilian consumers. Food Res. Int..

[14] Turkish Food Codex Honey Communiqué (Communiqué No: 2020/7) (2020). Republic of Turkey Ministry of Food, Agriculture and Livestock. Official Gazette No: 31107. https://www.resmigazete.gov.tr/eskiler/2020/04/20200422-13.htm.

[15] Venir E., Spaziani M., Maltini E. (2010). Crystallization in “Tarassaco” Italian honey studied by DSC. Food Chem..

[16] Scripca L., Amariei S. (2018). Research on honey crystalization. Rev. Chim..

[17] Vijayakumar K., Bhat N., Neethu T., Nayimabanu T., Nithin H. (2021). Periodical changes in quality parameters of honey during storage and processing. Int. J. Chem. Stud..

[18] Solayman M., Shapla U.M., Khalil I., Khalil M.I., Gan S.H., Goh B.H. (2023). Furfural and Hydroxymethylfurfural. Honey: Composition and Health Benefits.

[19] Shapla U.M., Solayman M., Alam N., Khalil M.I., Gan S.H. (2018). 5-Hydroxymethylfurfural (HMF) levels in honey and other food products: Effects on bees and human health. Chem. Cent. J..

[20] Tosi E., Ciappini M., Re E., Lucero H. (2002). Honey thermal treatment effects on hydroxymethylfurfural content. Food Chem..

[21] The Council of the European Union (2002). Council Directive 2001/110/EC of 20 December 2001 relating to honey. Off. J. Eur. Communities L 10/47.

[22] Modi B., Timilsina H., Bhandari S., Achhami A., Pakka S., Shrestha P., Kandel D., Gc D.B., Khatri S., Chhetri P.M. (2021). Current trends of food analysis, safety, and packaging. Int. J. Food Sci..

[23] Pasias I.N., Raptopoulou K.G., Makrigennis G., Ntakoulas D.D., Lembessis D., Dimakis V., Katsinas R., Proestos C. (2022). Finding the optimum treatment procedure to delay honey crystallization without reducing its quality. Food Chem..

[24] Seraglio S.K.T., Bergamo G., Molognoni L., Daguer H., Silva B., Gonzaga L.V., Fett R., Costa A.C.O. (2021). Quality changes during long-term storage of a peculiar Brazilian honeydew honey: “Bracatinga”. J. Food Compos. Anal..

[25] Martínez R.A., Schvezov N., Brumovsky L.A., Puccoarello Roman A.B. (2018). Influence of temperature and packaging type on quality parameters and antimicrobial properties during Yateí honey storage. Food Sci. Technol..

[26] AOAC (1990). Official Methods of Analysis of the Association of Official Analytical Chemists.

[27] International Honey Commission Harmonised methods of the International Honey Commission. 2009, 1–63. http://www.bee-hexagon.net/files/file/fileE/IHCPapers/IHC-methods_2009.pdf.

[28] Cunniff P., AOAC (1995). Official Methods of Analysis of AOAC International.

[29] Ramay M.S., Yalcin S. (2020). Effects of supplemental pine needles powder (*Pinus brutia*) on growth performance, breast meat composition, and antioxidant status in broilers fed linseed oil-based diets. Poult. Sci..

[30] Goth L. (1991). A simple method for determination of serum catalase activity and revision of reference range. Clin. Chim. Acta..

[31] Singleton V.L., Rossi J.A. (1965). Colorimetry of total phenolics with phosphomolybdic-phosphotungstic acid reagents. Am. J. Enol. Vitic..

[32] Singleton V.L. (1999). Lamuela-Raventos: Analysis of total phenoles and other oxidation substartes and antioxidants by means of folin-ciocalteu reagent. Methods Enzymol..

[33] Juan-Borrás M., Periche A., Domenech E., Escriche I. (2015). Routine quality control in honey packaging companies as a key to guarantee consumer safety. The case of the presence of sulfonamides analyzed with LC-MS-MS. Food Control.

[34] Minhas S. (2010). Nutritional Storage and Value Addition Studies on Raw and Heat Processed Honey.

[35] da Silva P.M., Gonzaga L.V., Biluca F.C., Schulz M., Vitali L., Micke G.A., Costa A.C.O., Fett R. (2020). Stability of Brazilian Apis mellifera L. honey during prolonged storage: Physicochemical parameters and bioactive compounds. LWT.

[36] Prica N., Baloš M.Ž., Jakšić S., Mihaljev Ž., Kartalović B., Babić J., Savić S. (2014). Moisture and acidity as indicators of the quality of honey originating from Vojvodina region. Arch. Vet. Med..

[37] Monggudal M., Radzi M., Ismail M., Ismail W.W. Effect of six month storage on physicochemical analysis and antioxidant activity of several types of honey. Proceedings of the IOP Conference Series: Materials Science and Engineering.

[38] Terrab A., Díez M.J., Heredia F.J. (2002). Characterisation of Moroccan unifloral honeys by their physicochemical characteristics. Food Chem..

[39] European Commission Opinion of the Scientific Committee on Veterinary Measures Relating to Public Health on Honey and Microbiological Hazards. https://food.ec.europa.eu/document/download/7488a863-4179-444a-8eff-72b137ae0240_en?filename=sci-com_scv_out53_en.pdf.

[40] Faleye F., Soyinka J., Omoniyi S., Popoola O., Adegbola A., Akinpelu B., Adekeye D. (2021). Physicochemical properties, antioxidant and antimicrobial activities of Nigerian Polyfloral honeys. J. Pharmacogn. Phytochem..

[41] Bhalchandra W., Joshi M.A., Jawalkar N. (2022). Effect of storage on various honey quality parameters of Apis mellifera honey harvested from Kannad region, Aurangabad. J. Pharmacogn. Phytochem..

[42] DeMera J.H., Angert E.R. (2004). Comparison of the antimicrobial activity of honey produced by Tetragonisca angustula (Meliponinae) and Apis mellifera from different phytogeographic regions of Costa Rica. Apidologie.

[43] Sanz S., Gradillas G., Jimeno F., Perez C., Juan T. (1995). Fermentation problem in Spanish North-Coast Honey. J. Food Prot..

[44] Silva M.S., Rabadzhiev Y., Eller M.R., Iliev I., Ivanova I., Santana W.C. (2017). Microorganisms in honey. Honey Anal..

[45] Kamal A., Raza S., Rashid N., Hameed T., Gilani M., Qureshi M.A., Nasim K. (2002). Comparative study of honey collected from different flora of Pakistan. Online JB Sci..

[46] Pirdawd H.K. (2022). Physiochemical Analysis of Honey Produced Around the City of Erbil (Iraq). Harran University Graduate School of Natural and Applied Sciences. MSc Thesis, Şanlıurfa. http://hdl.handle.net/11513/3266.

[47] Habib H.M., Al Meqbali F.T., Kamal H., Souka U.D., Ibrahim W.H. (2014). Physicochemical and biochemical properties of honeys from arid regions. Food Chem..

[48] Brugnerotto P., Fuente-Ballesteros A., Martín-Gómez B., María Ares A., Valdemiro Gonzaga L., Fett R., Carolina Oliveira Costa A., Bernal J. (2024). Free amino acid profile in Mimosa scabrella honeydew honey from Brazil and chemometric analysis for geographical discrimination. Food Res. Int..

[49] Hermosín I., Chicon R.M., Cabezudo M.D. (2003). Free amino acid composition and botanical origin of honey. Food Chem..

[50] Zhang G.Z., Tian J., Zhang Y.Z., Li S.S., Zheng H.Q., Hu F.L. (2021). Investigation of the maturity evaluation indicator of honey in natural ripening process: The case of rape honey. Foods.

[51] Chua L.S., Adnan N.A. (2014). Biochemical and nutritional components of selected honey samples. Acta. Sci. Pol. Technol. Aliment.

[52] Gheldof N., Wang X.H., Engeseth N.J. (2002). Identification and quantification of antioxidant components of honeys from various floral sources. J. Agric. Food Chem..

[53] Lawag I.L., Islam M.K., Sostaric T., Lim L.Y., Hammer K., Locher C. (2023). Antioxidant activity and phenolic compound identification and quantification in Western Australian Honeys. Antioxidants.

[54] Alvarez-Suarez J.M., Giampieri F., Battino M. (2013). Honey as a source of dietary antioxidants: Structures, bioavailability and evidence of protective effects against human chronic diseases. Curr. Med. Chem..

[55] Fadzil M.A.M., Mustar S., Rashed A.A. (2023). The potential use of honey as a neuroprotective agent for the management of neurodegenerative diseases. Nutrients.

[56] Alaerjani W.M.A., Abu-Melha S., Alshareef R.M.H., Al-Farhan B.S., Ghramh H.A., Al-Shehri B.M.A., Bajaber M.A., Khan K.A., Alrooqi M.M., Modawe G.A. (2022). Biochemical reactions and their biological contributions in honey. Molecules.

[57] Namiki M. (1988). Chemistry of Maillard reactions: Recent studies on the browning reaction mechanism and the development of antioxidants and mutagens. Adv. Food Res..

[58] Wang X., Gheldof N., Engeseth N. (2004). Effect of processing and storage on antioxidant capacity of honey. J. Food Sci..

[59] Kedzierska-Matysek M., Stryjecka M., Teter A., Skalecki P., Domaradzki P., Florek M. (2021). Relationships between the content of phenolic compounds and the antioxidant activity of Polish honey varieties as a tool for botanical discrimination. Molecules.

[60] Koç A.U., Atakan Y., Küçüker H., Küçüker H. (2023). Some physicochemical characteristics of Denizli thyme (*Origanum onites*) honey. Adnan Menderes Üniv. Zir. Fak. Derg..

[61] Kıvrak Ş., Kivrak I., Karababa E. (2016). Characterization of Turkish honeys regarding of physicochemical properties, and their adulteration analysis. Food Sci. Technol..

[62] Meda A., Lamien C.E., Romito M., Millogo J., Nacoulma O.G. (2005). Determination of the total phenolic, flavonoid and proline contents in Burkina Fasan honey, as well as their radical scavenging activity. Food Chem..

[63] Socha R., Juszczak L., Pietrzyk S., Gałkowska D., Fortuna T., Witczak T. (2011). Phenolic profile and antioxidant properties of Polish honeys. Int. J. Food Sci. Technol..

