# Supplementary material for: Effects of Different Packaging Types and Storage Periods on Physicochemical and Antioxidant Properties of Honeys
Source: Foods. 2024 Nov 10;13(22):3594. doi: 10.3390/foods13223594 (PMC11593547; doi:10.3390/foods13223594)
Supplement: Supplementary file 1 [file foods-13-03594-s001.zip › foods-3217343-SI.pdf]

Supplementary File. Spearman rho correlation analysis of physicochemical and antioxidant parameters at the initial, the 6th and 12th month of the storage in the groups.

	Diastase activity	HMF	Total sugar	Moisture	pH	Electrical conductivity	Free acidity	TAS	TOS	OSI	CAT	TPC
**Baseline**												
Proline	0.46*	0.14	-0.12	-0.58**	0.42*	0.39*	-0.14	0.35	0.20	-0.01	0.06	0.51**
Diastase activity		-0.49*	0.18	-0.27	0.82***	0.82***	0.48*	0.00	0.06	0.03	0.04	0.13
HMF			-0.03	-0.18	-0.40*	-0.54**	-0.71***	0.18	-0.30	-0.34	0.42*	0.28
Total sugar				-0.07	0.03	-0.16	0.25	-0.30	-0.80***	-0.75***	-0.10	-0.09
Moisture					-0.32	-0.14	-0.15	0.07	0.08	0.08	0.17	-0.27
pH						0.70***	0.56**	-0.10	0.12	0.22	0.00	-0.02
Electrical conductivity							0.37	0.19	0.38*	0.24	0.08	0.30
Free acidity								-0.50**	-0.04	0.21	-0.35	-0.44*
TAS									0.39*	-0.06	-0.06	0.64***
TOS										0.83***	-0.11	0.27
OSI											-0.08	-0.08
CAT												0.02
**In the 6th month of storage**												
Proline	0.59**	-0.06	-0.15	-0.40*	0.44*	0.36	-0.51**	0.61**	0.12	-0.29	0.31	0.51**
Diastase activity		-0.73***	-0.41*	-0.10	0.63***	0.66***	-0.23	0.39*	0.06	-0.18	0.41*	0.31
HMF			0.47*	-0.08	-0.42*	-0.64***	-0.07	-0.14	-0.12	-0.05	-0.32	-0.14
Total sugar				0.43*	-0.05	-0.37	0.14	-0.32	-0.63***	-0.41*	-0.10	-0.19
Moisture					-0.28	-0.21	0.25	-0.31	-0.09	0.14	-0.10	-0.16
pH						0.61**	-0.11	0.19	-0.29	-0.31	0.26	0.24
Electrical conductivity							-0.04	0.45*	0.13	-0.12	0.10	0.47*
Free acidity								-0.50**	-0.10	0.25	-0.14	-0.51**
TAS									0.34	-0.35	0.15	0.83***
TOS										0.70***	-0.05	0.14
OSI											-0.14	-0.45*
CAT												0.13
**In the 12th month of storage**												
Proline	0.70***	-0.01	-0.26	-0.36	0.37	0.33	-0.44*	0.62**	0.07	-0.53**	0.22	0.74***
Diastase activity		-0.55**	-0.60**	-0.21	0.31	0.55**	-0.41*	0.65***	0.20	-0.49**	0.45*	0.79***
HMF			0.63***	-0.09	-0.43*	-0.68***	-0.03	-0.14	-0.15	0.07	-0.22	-0.37
Total sugar				0.31	-0.22	-0.39*	0.11	-0.38	-0.50**	-0.03	-0.28	-0.46*
Moisture					-0.19	-0.23	0.18	-0.32	-0.04	0.19	-0.26	-0.39*
pH						0.75***	-0.07	0.04	-0.20	-0.20	0.07	0.43*

Electrical conductivity	0.04	0.30	0.03	-0.29	0.17	0.52**
Free acidity		-0.38	0.23	0.59**	-0.25	-0.46**
TAS			0.38	-0.57**	0.24	0.55**
TOS				0.49**	0.02	-0.12
OSI					-0.32	-0.64***
CAT						0.44*

*p<0.05; **p<0.01; ***p<0.001; HMF: hydroxymethylfurfural, TAS: total antioxidant status, TOS: total oxidant status, OSI: oxidative stress index, CAT: catalase; TPC: total phenolic content
